# Therapeutic Potential of Chemically Modified miR-489 in Triple-Negative Breast Cancers

**DOI:** 10.3390/cancers12082209

**Published:** 2020-08-07

**Authors:** Young Hwa Soung, Heesung Chung, Cecilia Yan, Andrew Fesler, Hyungjin Kim, Eok-Soo Oh, Jingfang Ju, Jun Chung

**Affiliations:** 1 Department of Pathology, Renaissance School of Medicine, Stony Brook University, Stony Brook, NY 11794, USA; younghwa.song@stonybrookmedicine.edu (Y.H.S.); heesung.chung@stonybrook.edu (H.C.); cecilia.yan@stonybrook.edu (C.Y.); Andrew.Fesler@stonybrook.edu (A.F.); Jingfang.Ju@stonybrookmedicine.edu (J.J.); 2 Department of Life Sciences, Ewha Womans University, Seoul 03760, Republic of Korea; OhEs@ewha.ac.kr; 3 Department of Pharmacological Sciences, Renaissance School of Medicine, Stony Brook University, Stony Brook, NY 11794, USA; hyungjin.kim@stonybrook.edu

**Keywords:** ARRDC3, miR-489, CMM489, chemoresistance, triple-negative breast cancers

## Abstract

Triple-negative breast cancers (TNBCs) lack ER, PR and her2 receptors that are targets of common breast cancer therapies with poor prognosis due to their high rates of metastasis and chemoresistance. Based on our previous studies that epigenetic silencing of a potential metastasis suppressor, arrestin domain-containing 3 (ARRDC3), is linked to the aggressive nature of TNBCs, we identified a sub-group of tumor suppressing miRNAs whose expressions were significantly up-regulated by ARRDC3 over-expression in TNBC cells. Among these tumor suppressing miRs, we found that miR-489 is most anti-proliferative in TNBC cells. miR-489 also blocked DNA damaging responses (DDRs) in TNBC cells. To define the mechanism by which miR-489 inhibits TNBC cell functions, we screened the potential target genes of miR-489 and identified MDC-1 and SUZ-12 as novel target genes of miR-489 in TNBC cells. To further exploit the therapeutic potentials of miR-489 in TNBC models, we chemically modified the guide strand of miR-489 (CMM489) by replacing Uracil with 5-fluorouracil (5-FU) so that tumor suppressor (miR-489) and DNA damaging (5-FU) components are combined into a single agent as a novel drug candidate for TNBCs. Our studies demonstrated that CMM489 shows superior effects over miR-489 or 5-FU in inhibition of TNBC cell proliferation and tumor progression, suggesting its therapeutic efficacy in TNBC models.

## 1. Introduction

TNBCs are an aggressive subset of breast tumors lacking estrogen receptor (ER), progesterone receptor (PR) and HER2 receptors with highly limited actionable molecular drug targets [1,2,3]. As these three receptors are commonly targeted for breast cancer therapy, TNBCs, unfortunately, exclude effective targeted therapeutic options and the standard of TNBC care remains chemotherapy [4,5]. The worst prognosis of TNBCs among all subtypes of breast cancer relates to their heterogeneity with high rates of metastasis to lung, brain and liver and chemoresistance [6]. Heterogeneity of TNBCs is often linked to genetic diversity caused by deficiencies in tumor suppressor genes that control DNA damaging responses (DDR) or defects in gatekeeper tumor suppressor genes that induce cell cycle arrest in response to DNA damages [7,8]. Although defects in DDR induce cancer development, they also provide cancer-specific vulnerabilities that can be therapeutically targeted because cancers with DDR defects tend to depend on an alternative DDR repair system to respond to genotoxic stress [9]. For this reason, targeting DDR defects through synthetic lethality (impairment of more than two DDR component resulting in cell death) emerges as a promising strategy by developing novel DDR inhibitors (DDRi) in cancer treatment including TNBCs [9].

Our previous studies and others have demonstrated that arrestin domain containing 3 (ARRDC3), a metastasis and tumor suppressor, is epigenetically silenced in metastatic TNBC cells [10], and levels of ARRDC3 in TNBC tumor tissues are significantly lower than those from benign tissues. Over-expression of ARRDC3 or treatment of small molecule compounds such as selinexor restored the expression of ARRDC3 in TNBC cells and effectively inhibited functions essential for TNBC progression in vitro and in vivo [11]. These outcomes provided the rationale of therapeutic targeting of ARRDC3 in TNBC models. In an effort to define the metastasis and chemoresistance suppressing mechanism of ARRDC3, we studied the relationship between ARRDC3 and potential tumor suppressing microRNAs (miRNAs) important in controlling metastatic potentials of TNBCs because regulation of miRNA expression has been implicated in multiple aspects of cancer progression [12,13,14,15]. We recently found that tumor suppressing activities of ARRDC3 are mainly mediated by tumor suppressing miRNAs [16]. Among these miRNAs, we demonstrated that miR-200b contributes to ARRDC3-mediated anti-metastatic functions such as reversal of EMT (Epithelial to Mesenchymal transition) phenotypes and inhibition of cell motility [16]. In the current studies, we decided to focus on the other ARRDC3-regulated miRNA, miR-489, based on its therapeutic potential associated with anti-neoplastic activities [17]. Levels of both ARRDC3 and miR-489 expression were shown to be significantly lower in metastatic and mesenchymal subtype TNBC tissues over adjacent normal tissues, suggesting their clinical relevance [11,17].

Our current studies suggest that miR-489 possesses a great therapeutic potential by blocking proliferation and DNA damaging responses in TNBC cells. We further exploited the therapeutic potential of miR-489 by chemically modifying the guide strand of miR-489 (CMM489) through replacing Uracil with 5-FU. Such modification allows the combination of tumor suppressing (through miR-489) and DNA damaging (through 5-FU) components to be combined into a single agent as a novel therapeutic molecule for treating TNBCs. Our in vitro and in vivo outcome suggests a superior therapeutic efficacy of CMM489 without any observable side effects. As a result, there is a great potential of CMM489 as a novel therapeutic option for TNBCs that currently has no effective targeted therapies merits consideration.

## 2. Results

### 2.1. MiR-489 Mediates Anti-Proliferative and DNA Damaging Response Blocking Function of ARRDC3 in TNBC Cells

**Figure 1:**
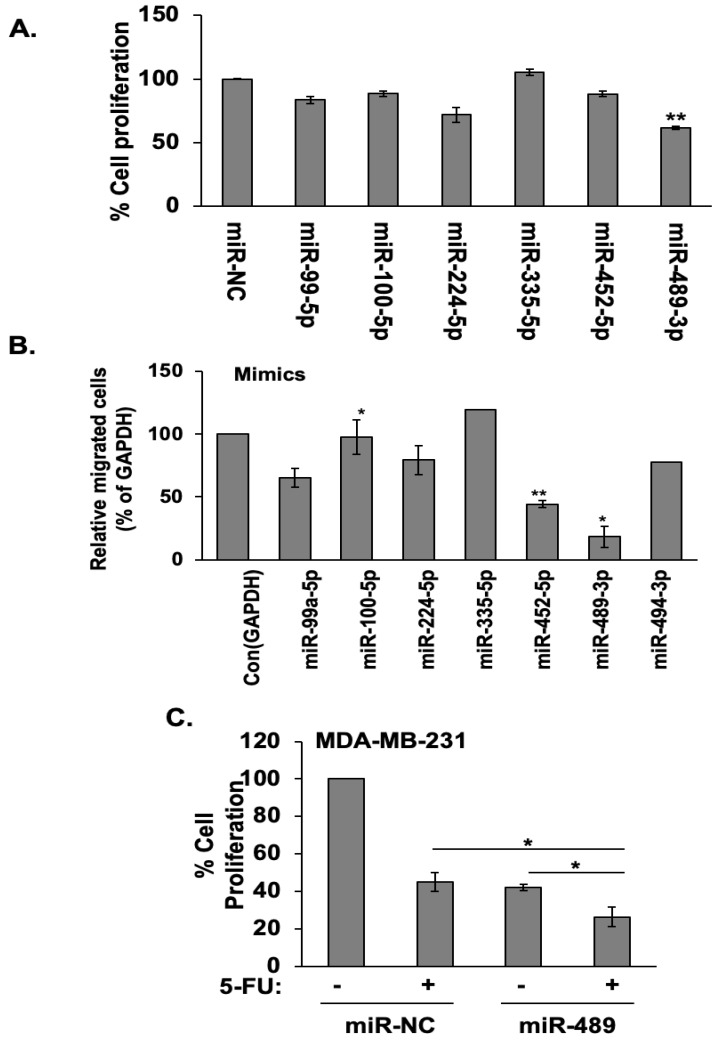

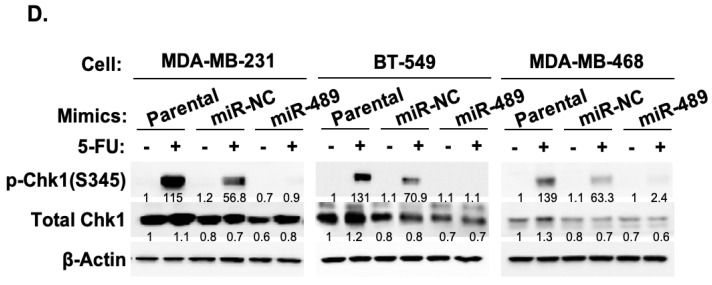
miR-489 mediates anti-proliferative and DNA damaging blocking functions of ARRDC3 in triple-negative breast cancer (TNBC). (**A**) MDA-MB-231 cells transfected with indicated miRNA mimics were incubated for 24, 48 and 96 hrs. Proliferation of these cells was measured by MTT assay. Data are represented as mean ± SD (*n* = 3). ** *p* < 0.01 compared with the control. (**B**) Cell migration assay of MDA-MB-231 cells with indicated miRNA mimics. Column, mean from three independent experiments; bars, SD. The statistical analysis was done using Student’s *t* test. * *p* < 0.05, ** *p* < 0.01. (**C**) MDA-MB-231 cells transfected with miR-negative control (miR-NC) and miR-489 mimics were incubated for 48 h, followed by with or without treatment of 5 μM 5-FU for 72 hrs. Proliferation of these cells was measured by MTT assay. Column, mean from three independent experiments; bars, SD. * *p* < 0.05; compared with the control. (**D**) Western blotting analysis p-Chk1 (S345), total Chk1 or β-Actin from lysates of MDA-MB-231, MDA-MB-468 and BT-549 TNBC cells transfected with or without miR-NC or miR-489, followed by with or without treatment of 5 μM 5-FU for 48 h. β-actin was used as loading control. Densitometric analysis was performed using Image J. The number shown underneath the band image represents the fold change compared with control. Representative results were obtained from three independent experiments. More details of western bolts, please view at the Supplementary Materials.

Our recent studies identified a group of tumor suppressing miRNAs whose expression is up-regulated 1.5-fold or higher by over-expression of ARRDC3 in TNBC cells [16]. Among these miRNAs, miR-489 was most effective in inhibiting the proliferation (Figure 1A and Figure S1) and cell motility (Figure 1B) of TNBC cells. Based on our recent studies that ARRDC3 sensitizes TNBC cells to DNA damaging agents [16], we tested whether inhibition of DNA damaging responses relates to the anti-cancer mechanism of miR-489 in TNBC cells. As shown in Figure 1C, expression of miR-489 in MDA-MB-231 cells sensitizes these cells to DNA damaging agents such as 5-FU. To further investigate this mechanism, we measured the phosphorylation levels of Chk1 in three TNBC cell lines upon treatment with control-miR or miR-489 with or without treatment of -5FU (Figure 1D). We measured the activation status of Chk1 by measuring phosphorylation levels at S345 because Chk1 activation involves ATM/ATR-mediated phosphorylation at S317/S345 and occurs in response to blocked DNA replication and certain genotoxic stress such as 5-FU treatment [18]. Chk1 phosphorylation levels at S345 are dramatically elevated upon treatment of DNA damaging agent 5-FU in three different TNBC cell lines. miR-489 expression efficiently blocked elevation of p-Chk1 at S345 by 5-FU, indicating its inhibitory function of DDR in all three TNBC cell lines (Figure 1C).

### 2.2. MDC1 and SUZ12 Are Novel Target Genes of MiR-489

To further define the anti-cancer mechanism of miR-489, we next attempt to identify key miR-489 target genes that mediate the blockade of DNA damaging responses in TNBC cells. We used the miRDB database (http://mirdb.org/miRDB) to predict the target genes for miR-489. A total of 398 miR-489 target genes were identified. Among them, 22 putative target genes with the probability of interaction (according to miRDB) given as a target score of 90 or higher were selected. Reference-based analysis of these target genes based on their potential roles in regulating DNA damaging responses and cancer progression led us to select SUZ-12 (polycomb repressive complex 2) and MDC-1 (mediator of DNA-damage checkpoint 1) as key target genes of miR-489.

**Figure 2:**
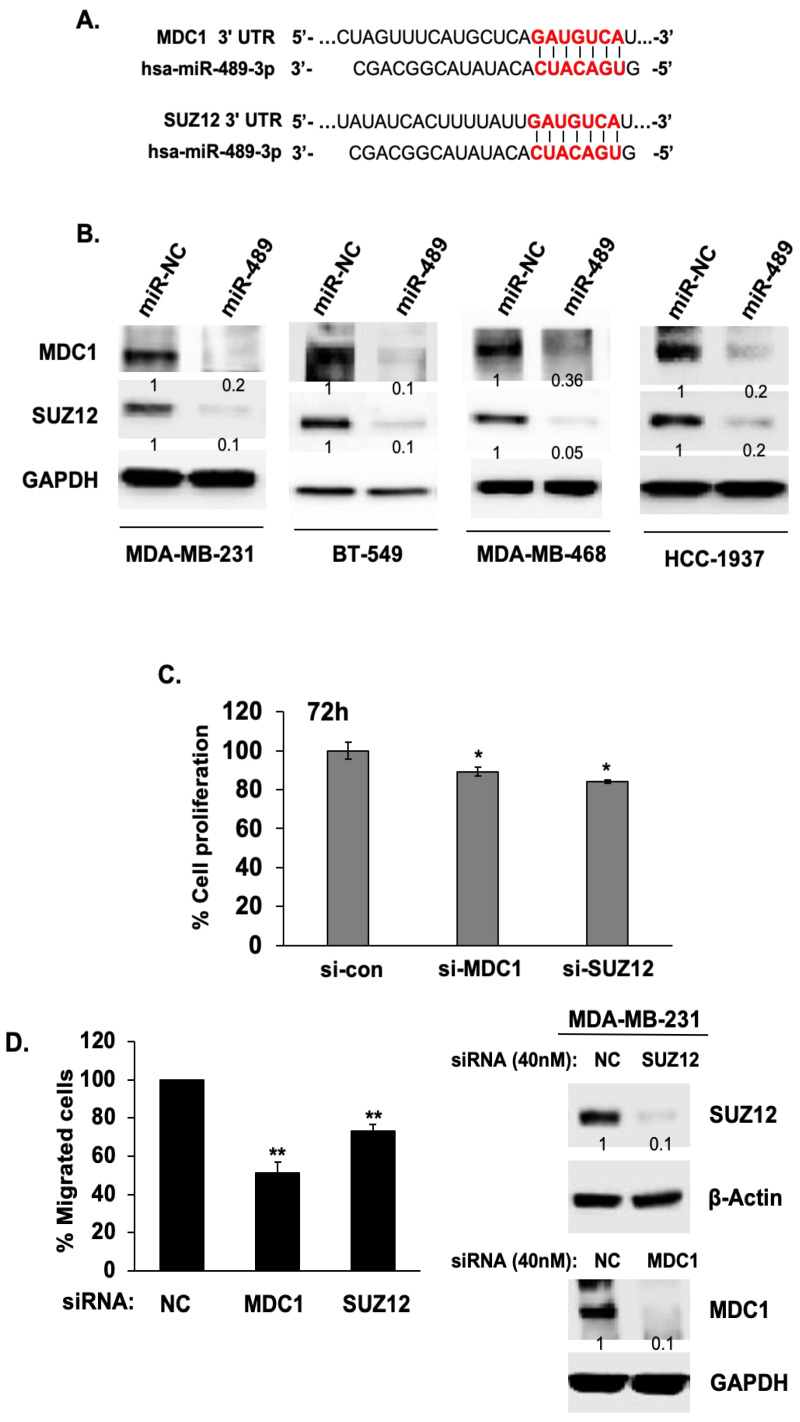

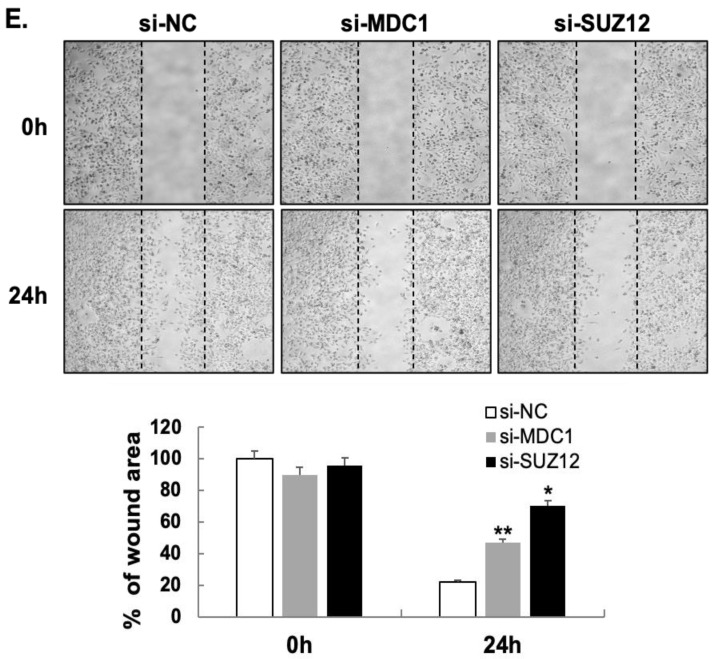
MDC1 and SUZ12 are novel target genes of miR-489 in TNBC cells. (**A**) miR-489 binding sites are found in the 3’ UTRs of MDC1 and SUZ12. (**B**) MDA-MB-231, HCC-1937, MDA-MB-468 and BT-459 cells were transfected with control miRNA (miR-NC) and miR-489. Protein levels from cell lysates were analyzed by Western blot assay with antibodies against MDC1 and SUZ12. GAPDH levels were used as loading control. Densitometric analysis was performed using Image J. Representative images were carried out at least three times. (**C**) MDA-MB-231 cells were transfected with 40 nM siRNA negative control (NC), siRNA MDC1 or siRNA SUZ12. The rates of proliferation were measured at 72 h upon transfection. Proliferation of these cells was measured by MTT assay. Column, mean from three independent experiments; * *p* < 0.05; compared with the control. (**D**) MDA-MB-231 cells were transiently transfected with either siRNA NC (as negative control), siRNA MDC1 or siRNA SUZ12. The cell migration was measured using a transwell cell motility assay and quantified by counting the cells (right panel). Column, mean from three independent experiments; bars, SD. ** *p* < 0.01 compared with the control. The knockdown was evaluated by Western blot assay with MDC1 and SUZ12 antibodies (left panel). β-actin and GAPDH were used as loading control. Densitometric analysis was performed using Image J. Representative images were carried out at least three times. (**E**) MDA-MB-231 cells transfected with siRNA NC, siRNA MDC 1 and siRNA SUZ12 were loaded into Ibidi’s culture-insert 2 well in μ-dish and allowed to adhere overnight. Cells that migrated into the wound area were captured by a phase contrast microscope at the indicated time points. Quantification of wound area was analyzed using Image J. Representative images were carried out at least three times. Column, mean from three independent experiments; bars, SD. ** *p* < 0.01, * *p* < 0.05. More details of western bolts, please view at the Supplementary Materials.

SUZ-12 and MDC-1 contain binding sites in their 3′ UTR, which implies that SUZ-12 and MDC-1 are target genes of miR-489 (Figure 2A). Western blotting analysis showed a reduction in SUZ-12 and MDC-1 protein levels when miR-489 is expressed in all three TNBC cell lines (Figure 2B). SUZ-12 has been identified at the breakpoints of a recurrent chromosomal translocation and may be involved in chromatin silencing [19] and has been shown to contribute to epithelial to mesenchymal transition in breast cancer [20]. MDC-1 has been identified as a prognostic marker for recurrence of early stage breast cancer [21] and its level is positively correlated with recurrence in breast cancer [22]. Knockdown of MDC1 and SUZ12 by siRNA transfection effectively reduced TNBC cell proliferation (Figure 2C), motility towards LPA (Figure 2D) and wound healing (Figure 2E) compared to those of the control cells. These results suggest that MDC1 and SUZ12 are key target genes of miR-489 and play important roles in mediating miR-489’s anti-cancer functions. As the expression of both MDC1 and SUZ12 has been implicated with metastatic breast cancers including TNBCs [22,23,24], defining their roles downstream of miR-489 will provide novel insights in designing therapeutic targeting in TNBCs.

### 2.3. Chemically Modified MiR-489 Mimics (CMM489) Have Enhanced Therapeutic Efficacy in TNBC Cells

**Figure 3:**
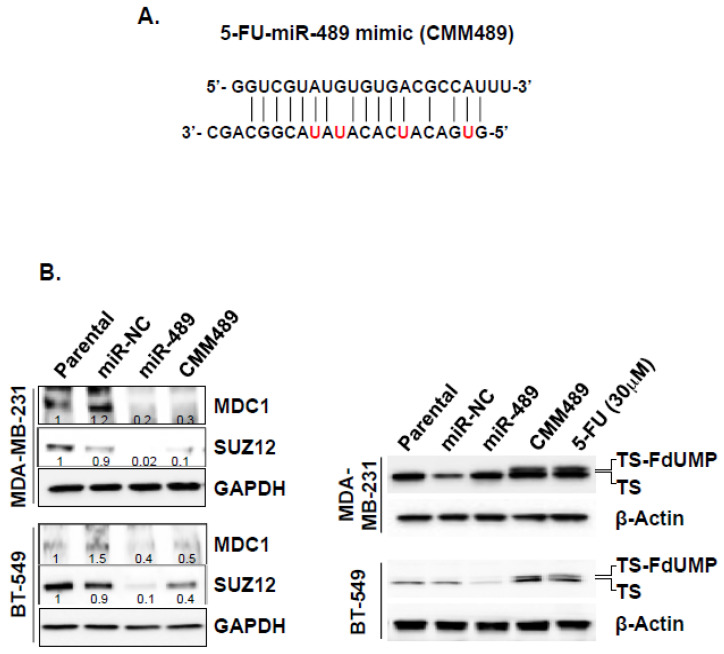

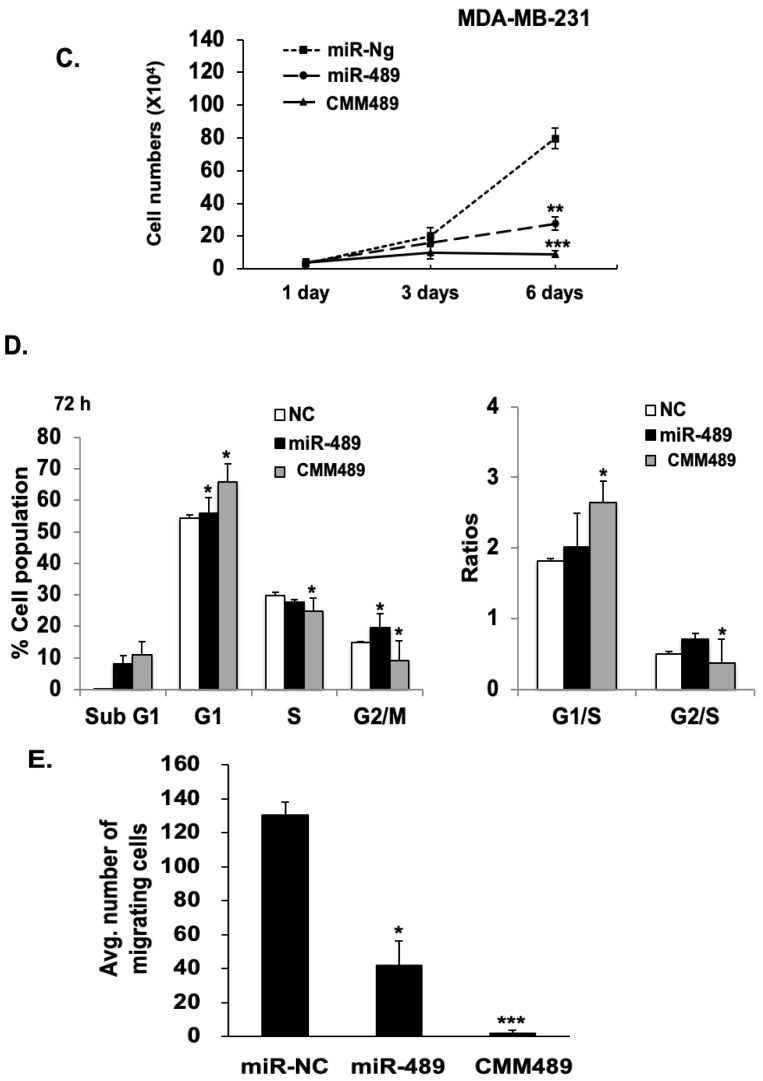

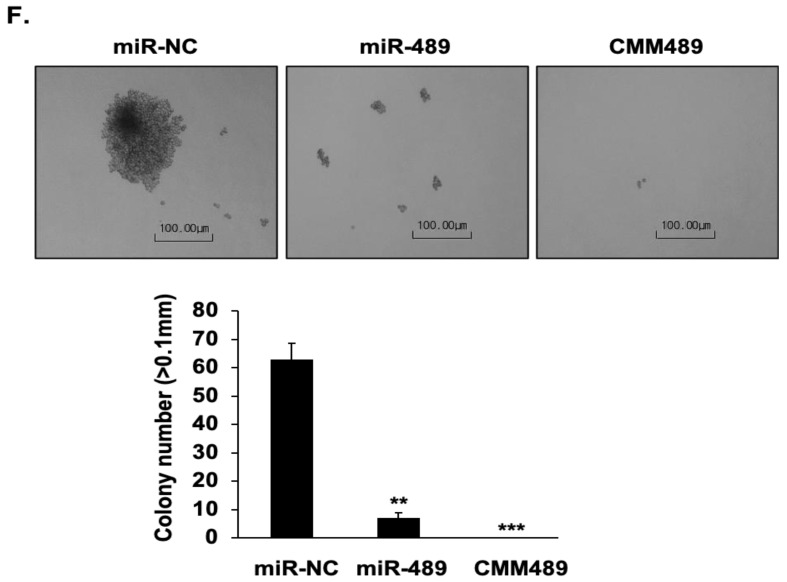
Enhanced therapeutic efficacies of CMM489 in TNBC cells. (**A**) Schematic description of CMM489. Uracil in miR-489 is replaced by 5-FU (red) in CMM489. (**B**) Western blotting analysis of SUZ-12, MDC-1 and GAPDH (left) and TS-FdUMP-TS and beta actin (right) from lysates of MDA-MB-231 and BT-549 cells transfected with none, miR-NC, miR-489, CMM489 or treated with 30 μM 5-FU. Densitometric analysis was performed using Image J. Representative images were carried out at least three times. (**C**) The rate of proliferation was monitored by cell counting MDA-MB-231 cells transfected with 100 nM of indicated miRs using oligofectamine at 1–6 days. Column, mean from three independent experiments; bars, SD. ** *p* < 0.01, *** *p* < 0.001. (**D**) MDA-MB-231 cells transfected with the indicated miRNAs were stained with propidium iodidie (PI) and the percentage of cell population in each cell cycle phase was analyzed by flow cytometry. Representative images were selected from three independent experiments. All data are presented as the means ± SD. * *p* < 0.05. (**E**) Inhibition of cell motility in MDA-MB-231 cells transfected with miR-NC, miR-489 or CMM489 was measured using a transwell assay. The ability of cells to migrate toward 100nM LPA as chemoattractants was quantified by counting the cells per square milliliter using bright-field optics. Column, mean from three independent experiments; bars, SD. * *p* < 0.05, *** *p* < 0.001 compared with the control. (**F**) MDA-MB-231 cells expressing miR-NC, miR-489 and CMM489 were cultured in soft agar-containing growth medium after 10 days. Colony formation was captured at 10x magnification. Only cell colonies >0.1 mm in diameter were counted. Representative images were carried out at least 3 times. Scale bar: 100 μm. All data are presented as the means ± SD. ** *p* < 0.01, *** *p* < 0.001.

To enhance the stability and efficacy of miR-489 as a therapeutic agent, we chemically modified the guide strand of miR-489 (CMM489) by replacing U with 5-FU (Figure 3A). The rationale behind this chemical modification is that the “5-FU component” of CMM489 induces single-stranded breakage of DNA, whereas the “miR-489 component” of CMM489 inhibits DNA damaging responses (DDRs) to prevent DNA repair so that synthetic lethality can occur by a single therapeutic agent. CMM489 retains the target gene specificity of miR-489 as demonstrated by reducing MDC1 and SUZ12 expression and also possesses the 5-FU function as demonstrated by the detection of the thymidylate synthase FdUMP (TS-FdUMP) complex (Figure 3B). miR-489 expression levels were confirmed in MDA-MB-231 cells transfected with CMM489 (Figure S2).

To assess the synthetic lethality effects of the 5-FU component and miR-489 component of CMM489, various functions of TNBC cells were compared after treating the cells with either CMM489 or native miR-489 or control miR. Anti-proliferative effects of CMM489 in MDA-MB-231 cells were far more effective than native miR-489 (Figure 3C). Consistent with this result, CMM489 increases sub G1 and G1/S ratio, whereas miR-489 increases sub G1 and G2/S ratio, suggesting more effective cell cycle arrest by CMM489 over native miR-489 (Figure 3D). In TNBC cell motility using the transwell assay, miR-489 reduced cell motility by 70%, whereas CMM489 nearly completely inhibited the cell motility (over 90%) (Figure 3E). Finally, we measured anchorage-independent growth of TNBC cells using the colony formation assay. Both miR-489 and CMM489 effectively blocked colony formation while there is virtually no colony formation in CMM489-treated TNBC cells (Figure 3E). All these functional assays suggest the superior therapeutic efficacy of CMM489 over native miR-489 in TNBC cells.

### 2.4. Vehicle Free Delivery of CMM489

**Figure 4:**
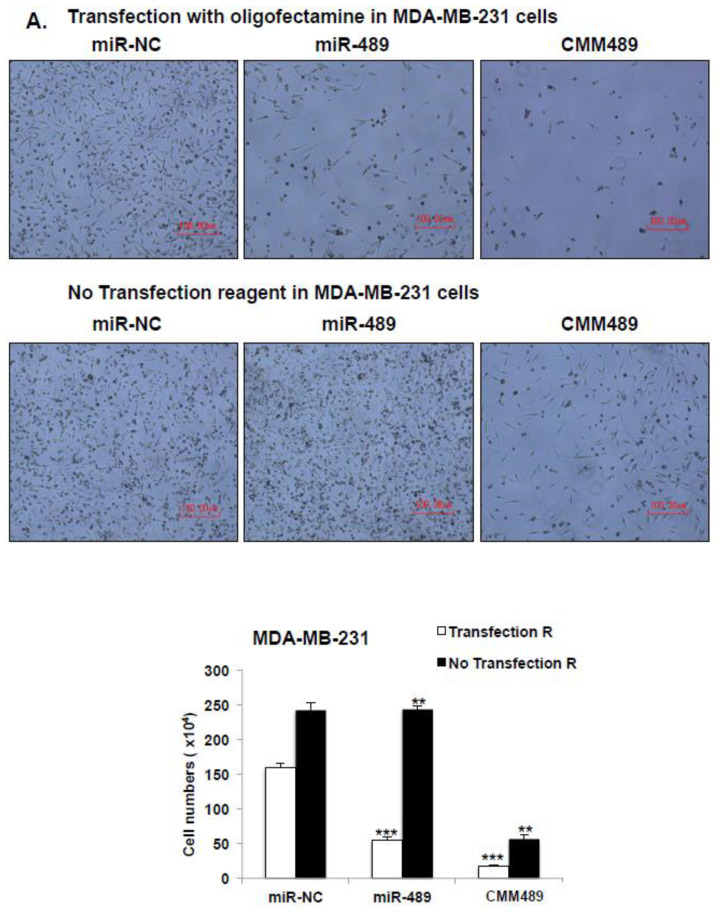

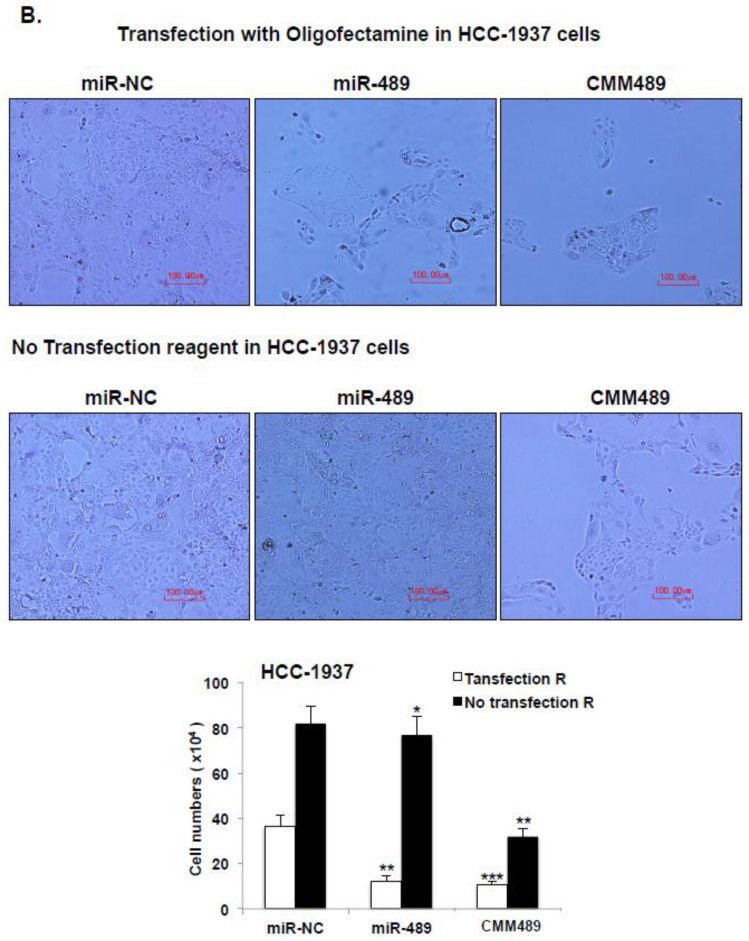

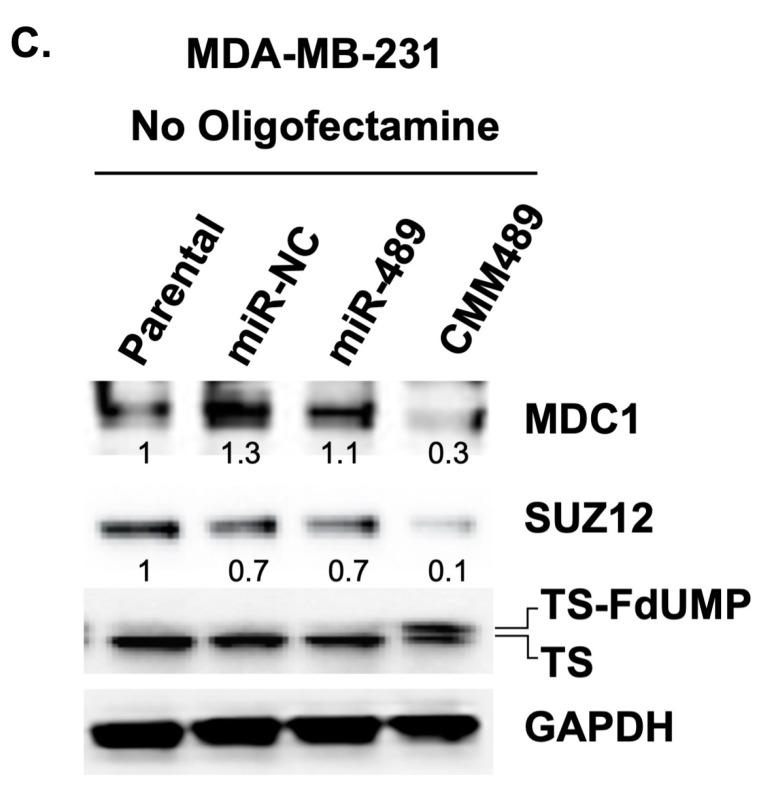
Delivery of 5-FU-miR-489 mimic (CMM489) does not require a transfection reagent. miR-negative control (NC), miR-489 and CMM489 were transfected into MDA-MB-231 cells (**A**) or HCC-1937 cells (**B**) with or without oligofectamine reagent. The density of cells was imaged at 6 days by phase contrast microscopy. Representative images were selected from three independent experiments. Scale bar: 100 μm. The cell numbers were counted using Trypan blue staining and a hematocytometer. Column, mean from three independent experiments; bars, SD. * *p* < 0.05, ** *p* < 0.01, *** *p* < 0.001. (**C**) Whole cell lysate from MDA-MB-231 cells transfected with the indicated miRNAs without oligofectamine reagent were analyzed by Western blot assay with antibodies against MDC1, SUZ12, TS-FdUMP and β-actin. Densitometric analysis was performed using Image J. Representative images were carried out at least 3 times.

Liposome-based transfection reagents such as Oligofectamine and lipofectamine are essential for the introduction of miRNAs into cells. Our previous studies demonstrated that 5-FU incorporation into tumor suppressing miRs miR-200b and miR- confers the ability of the 5-FU-200b mimic to enter into the cells without a delivery vehicle [16,25]. To test whether CMM489 can also be delivered to the cells without any transfection reagents, TNBC cells (MDA-MB-231 and HCC-1937) were transfected with miR-NC (negative control), miR-489, or CMM489 with or without a delivery vehicle (oligofectamine). We measured the effects of the expression of these miRs on TNBC cell proliferation by using Trypan blue staining and a hemocytometer for six days. The anti-proliferative activity of CMM489 is maintained even when CMM489 is incubated with TNBC cells without oligofectamine, whereas inhibitory effects of miR-489 were only observed in the presence of oligofectamine (Figure 4A,B). Consistent with these findings, CMM489, not miR-489, effectively suppresses the expression of the target genes such as MDC-1 and SUZ-12 and possesses 5-FU activity as confirmed by the formation of the thymidylate synthase FdUMP (TS-FdUMP) complex in the absence of oligofectamine (Figure 4C). These outcomes suggest that CMM489 could enter the cells and plays therapeutic roles in TNBC cells without transfection reagents.

### 2.5. CMM489 Inhibited Primary Tumor Growth and Metastasis in Metastatic Mouse Model

**Figure 5:**
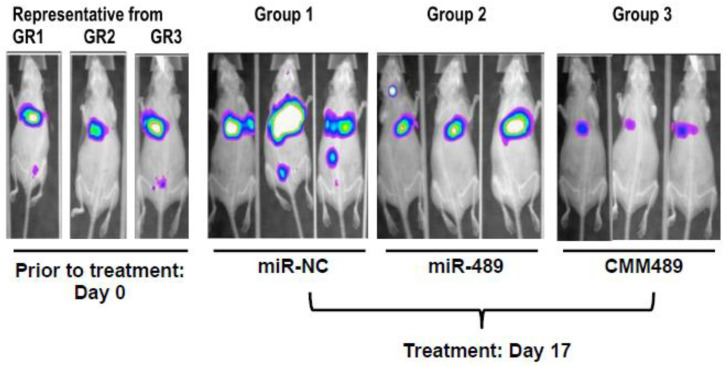
The effects of CMM489 treatment in vivo. Mice were implanted with MDA-MB-231 breast cancer cells expressing luciferase genes through tail vein injection. After establishing the tumor, 40 μg of miR-NC (group 1), miRNA-489 (group 2) or CMM 489 (group3) was delivered by using in vivo-jetPEI via tail vein injection every other day for 17 days. To monitor tumoral volume, mice were subjected to in vivo imaging. Image shows luminescence overlaid on X-ray. Tumor growth was visualized with D-luciferin. Representative images of animals from each group are shown.

We finally assessed the effects of CMM489 on tumor growth and metastasis in vivo by establishing tumors in NU/NU mice via tail vein injection with MDA-MB-231 (2 × 10^6^) cells expressing luciferase reporter. Starting one week after injection, mice were screened weekly using the In-vivo Xtreme imaging system (Bruker) to check for tumor growth. Once tumors were seen, mice were separated into groups treated via tail vein injection with 40 μg of indicated mi-RNA packaged with in vivo-jetPEI (Polyplus Transfection). The rationale to use in vivo-jetPEI to package CMM489 as a delivery vehicle is to minimize endosomal trapping of micoRNAs and to protect microRNAs from degradation in vivo. The in vivo-jetPEI has been demonstrated to be safe and effective in delivering nucleic acid-based therapeutics to multiple tissues including bone, brain, lung, liver and other major organs and tissues [26]. In addition, control miRs and native miRs cannot be delivered without a delivery vehicle unlike CMM489. Following treatment, mice were screened using the In-vivo Xtreme imaging system. Mice treated with control miR-NC showed spreading of secondary tumors in livers (Group 1, Figure 5). CMM489 efficiently reduced the primary tumor growth as well as metastatic tumor formation when delivered through tail vein injection (Group 3, Figure 5). Native miR-489 also prevented metastasis over the miR-NC-treated group, but efficiency of primary tumor growth inhibition was not as good as CMM489 (Group 2, Figure 5). These mice showed no signs of observed host toxicity such as weight and hair loss from CMM489 (Figure 5).

## 3. Discussion

In the current studies, we demonstrated that miR-489 is a key target gene of tumor suppressor ARRDC3. miR-489 plays a key role in mediating the anti-proliferative and DNA damaging response inhibition of ARRDC3, which we have seen in our previous studies. To further exploit the therapeutic potentials of miR-489, we chemically modified the guide strand of miR-489 (CMM489) by replacing internal U with 5-FU so that DNA damaging response inhibiting (through miR-489 and its target genes such as SUZ-12 and MDC-1) and DNA damaging (through 5-FU) components are combined into a single agent as a novel therapeutic molecule for study in TNBCs. Our preliminary studies demonstrated the superior inhibitory effects of CMM489 over native miR-489 in TNBC models in vitro and in vivo, suggesting that CMM489 is a novel therapeutic candidate for TNBCs.

With an enrichment of highly proliferative tumor and tumors with DNA damage repair (DDR) deficits, chemotherapy with DNA damaging agents (i.e., doxorubicin (DNA topoisomerase II inhibitor) and/or alkylating agents (platinum drugs)) remain the primary treatment modality for treatment of primary and metastatic TNBCs [6,7]. For example, TNBCs with BRCA deficiency demonstrate a greater overall sensitivity to DNA damaging agents and this sensitivity to genotoxic stress is a specific molecular vulnerability in TNBC [27]. As a result, DNA damaging response (DDR) inhibitors have been used in combination with chemotherapy for the treatment of TNBCs. However, not all TNBC subtypes respond well to DDR inhibitors (i.e., TNBCs without the germline BRCA 1/2 mutation) [9,28]. As three TNBC cell lines (MDA-MB-231, BT-549 and MDA-MB-468) that respond to CMM489 do not contain the BRAC1 or 2 mutation (Figure 1C), our studies suggest that CMM489 can be a novel DDR inhibitor candidate for TNBCs that do not possess inherent DDR deficiency.

The mechanism of the vehicle-free delivery of CMM489 remains a mystery. However, we consistently observed this phenomenon whenever we replaced uracil with 5-FU in the target micro-RNA [16,25]. While the vehicle-free delivery of the modified micro-RNA mimic requires further studies, multiple evidence supports that chemical modifications of oligonucleotides induced their ability to get into the cells without delivery vehicles via a process called “gymnosis” [29,30]. Nevertheless, the vehicle-free delivery of CMM489 further merits consideration of CMM489 as a potential therapeutic agent for TNBCs as it can remove potential toxicity associated with delivery tools.

To further enhance the stability and to avoid endosome trapping of CMM489 in vivo, we used in vivo-jetPEI (Polyplus Transfection) to boost the efficacy of CMM489 in vivo as in vivo-jetPEI has no toxicity at lower concentrations. in vivo-jetPEI has been used in human clinical trials as a gene therapy tool and showed its efficacy as well as no toxicity [31]. We should be able to achieve a good bi-standard effect of CMM489 to the neighboring tumor cells. As CMM489 did not induce weight and hair loss of the treated mice (Figure 5), we do not expect that delivery of CMM489 into non-tumor cells will trigger major toxicities. This could be due to the fact that the 5-FU portion of CMM489 is nearly 1000-fold lower (50 nM) than the reported IC50 values of 5-FU. We will attempt to further optimize CMM489 delivery in vivo and this will serve as the foundation for future therapeutics. The reduction of the treatment cycle from once every other day to once a week will be beneficial for human clinical trials. Overall, our studies will provide the basis for future clinical trials involving CMM489 as a novel therapy option for TNBCs.

## 4. Materials and Methods

### 4.1. Cell Lines and Reagents

All cell lines were obtained from ATCC. MDA-MB-231 and MDA-MB-436 breast cancer cells were maintained in growth medium (DMEM supplemented with 1 g/L glucose, L-glutamine, sodium pyruvate, 10% FBS and 1% penicillin/streptomycin). BT-549 and HCC-1937 cells were cultured in RPMI-1640 supplemented with 10% FBS and 1% penicillin/streptomycin. All cells were cultured in humidified incubators at 37 °C with 5% CO_2_.

SUZ12 (D39F6), Phospho-Chk1 (Ser345), Chk1 (2G1D5) and GAPDH antibodies were purchased from Cell Signaling (Danvers, MA, USA). MDC1 antibody was obtained from Novus. ITGB4 (H-101) and β-actin (clone C-11) antibodies were purchased from Santa Cruz Biotechnology (Santa, Dallas, TX, USA). Thymidylate Synthase (clone TS106) antibody was purchased from EMD Millipore (Burlington, MA, USA).

### 4.2. MiRNA Preparation and Transfection

Pre-miR precursor miR-489-3p and pre-miR negative control precursor (NC #2, miR-NC) were purchased from Ambion (Invitrogen, Carlsbad, CA, USA). miRIDIAN miRNA-489-3p mimics were purchased from Dharmacon (Lafayette, CO, USA). To generate chemically modified 5-FU-miR-489 mimics (CMM489), the guide strand RNA was modified with 5-fluorouridine in place of internal U. The passenger strand contains no modification. The equimolar concentration of oligomers was combined in a single tube. The 2-ACE protecting groups of RNA oligonucleotides were removed by acid-catalyzed hydrolysis in the deprotection step. The deprotected oligoes were annealed to produce RNA duplexes (called mimics). For transfection, cells were seeded on the 6-well plate and transfected with miRNAs or CMM489 using oligofectamine (Invitrogen-Life Technologies, Carlsbad, CA, USA), according to the manufacturer’s instructions. For oligofectamine-free transfection, miRNAs were diluted in Opti-MEM and then added into cells.

### 4.3. Identification of MiR-489 Target Genes

The targets of miR-489 were predicted by three web databases including TargetScan v.7.1. (http://www.targetscan.org/vert_71/), miRDB (http://mirdb.org/miRDB) and miRSystem (http://mirsystem.cgm.ntu.edu.tw). According to miRDB database, putative target genes were evaluated based on the probability of interaction given as a target score >90. miR-489 target genes selected from at least three databases were further validated using mRNA microarray expression profile data (Affymetrix U133 plus 2.0) to detect down- and up-regulated genes in TNBC cells as compared to ARRDC3 over-expressing cells. Significance analysis of microarrays (SAM) was conducted to determine differential mRNA expression. The data from Affymetrix microarray expression were analyzed by Mann–Whitney U Test and *p* < 0.05 was considered as significant.

### 4.4. SiRNA Transfection

siRNA transfection was performed using RNAiMAX (Invitrogen) according to the manufacturers’ instructions. MCD1 Silencer pre-designed siRNA (AM16709: 5′-GGAUCACACAAAGAUUAGAtt) and Silencer negative control #2 were purchased from Ambion, Life Technology (Austin, TX, USA); SUZ12 siRNA was provided by H Kim (Pharmacological Sciences, Stony Brook Medicine).

### 4.5. Western Blot Analysis

Cells were lysed using cold RIPA-EDTA buffer [50 mM Tris, pH 7.4; 150 mM NaCl; 1% NP-40; 0.5% sodium deoxycholate; 0.1% SDS; and 5 mM EDTA] containing 1 mM phenylmethylsulfonyl fluoride, 1 mM Na_3_VO_4_ and protease inhibitor (Thermo Scientific Pierce, Rockford, IL, USA). The protein concentrations were determined using the BCA protein assay kit (Thermo Scientific Pierce). The proteins were separated on 4% to 20% gradient SDS PAGE and transferred to PVDF membranes by using the Trans-Blot Turbo transfer system (Bio-Rad, Hercules, CA, USA). The blots were incubated with primary antibodies in TBS-T or TBS-T with 5% *w/v* nonfat dry milk for 4 °C and then with the appropriate secondary antibodies conjugated to IgG-horseradish peroxidase. Proteins were detected using the Clarity Western ECL blotting substrate (Bio-Rad). All bands were imaged with ChemiDoc Touch Imaging System (Bio-Rad).

### 4.6. Cell Viability by MTT Assay

Cells were seeded in 96-well plates with 100 μL media in triplicate and allowed to adhere overnight. The cells were treated with 5-FU at the concentrations indicated. After the treatment for 24, 48 or 72 h, viability was evaluated using the Kit-8 (Dojundo Molecular Technologies, Rockville, MD, USA) according to the manufacturer’s instructions. Absorption at 450 nm was determined using an iMark Microplate Reader (Bio Rad). IC_50_ values, representing the drug concentration causing 50% growth inhibition, were calculated using https://www.aatbio.com/tools/ic50-calculator.

### 4.7. Cell Cycle Assay

Cells plated on the 6-well plate were transfected with miRNAs. After incubation for 48 h, cells were collected by trypsinization, washed with PBS and fixed in 66% ethanol at 4 °C for 1 h. Cells were washed with PBS and stained in 200 μL of propidium iodide (PI) and RNase stating solution (PI Flow Cytometry kit for cell cycle analysis, Abcam, Cambridge, MA, USA), followed by incubation at 37 °C for 20 min. Cell cycle distribution was examined using the BD FACSCalibur flow cytometer (BD Bioscience) and analyzed using the ModFit LT v3.3 software. Each sample was analyzed in triplicate.

### 4.8. Cell Motility Assay

Cell motility assays were performed by a transwell cell culture chamber of 8 μm pore size (Costar-Falcon, Teterboro, NJ, USA) according to the standard procedure. Transwell inserts were coated with collagen I (15 μg/mL) overnight at 4 °C. After washing the inserts with PBS the next day, cells were added to the upper chamber of each well. LPA (Lysophosphatidic acid; 100 ng/mL) was added to the lower chambers as a chemoattractant. The chambers were incubated for 2 h at 37 °C with 10% CO_2_. The cells that did not migrate through the pores were mechanically removed by cotton swab. The migrated cells on the lower surface of the membrane were fixed and stained with 0.2% crystal violet and counted. Assays were performed in triplicate and repeated three times.

### 4.9. Colony Formation Assay

MDA-MB-231 cells transfected with control (NC), miR-489 and 5-Fu-miR489 mimic were suspended in the top layer of DMEM (1 mL) containing 0.35% low-melt agarose (ISC Bioexpress, Kaysville, UT, USA) and then the top layer was overlaid on DMEM (2 mL) containing 0.75% agar in 6-well plates. The cells were fed twice per week with 0.5 mL DMEM. After 3 weeks, colonies larger than 0.1 mm in diameter were counted per well by using bright-field optics. The images of colonies were acquired by a microscope and digital camera (Nikon). The average number of colonies was obtained from counting triplicate wells.

### 4.10. Wound-Healing Assay

MDA-MB-231 cells were loaded into each well of a 35 mm culture μ-dish (Ibidi, Bonn, Germany) at 2 × 10^5^ cells/well and incubated overnight at 37 °C, allowing cells to adhere and spread. The insert was removed and attached cells were washed twice with 1 × PBS. The progression of wound closure and the cells that migrated into empty spaces of the wounded area were determined under an inverted phase-contrast microscope with a distal camera (Nikon). Images were captured at indicated time points. Wound-healing assays were carried out in triplicates.

### 4.11. Mouse Xenograft and Imaging

All animal procedures were performed by Pharma models LLC (Marlborough, MA, USA). All experiments were carried out in accordance with the guidelines of the guide for animal welfare and with the approval of the IACUC. Fifteen (15) NU/NU mice were implanted with 2 × 10^6^ luciferase-expressing MDA-MB-231 cells. All mice were imaged once a week to check for tumor formation and growth. Tumors were measured on Mondays, Wednesdays and Fridays by measuring each tumor in 2 dimensions. Tumor location was noted, and signal intensity was quantified. When the desired florescence was attained (average radiance 1e3–1e4), treatment was started. For treatment with miRNAs in the breast cancer in vivo model, mice were treated with 40 ug vehicle control, miRNA-489 or CMM 489 via tail vein injection by using in vivo-jetPEI (polyplus-transfection SA, Illkirch, France) every other day for 17 days. After treatment, mice were imaged twice more, 1 and 2 weeks post-treatment to assess tumor size based on signal quantification. Mice were evaluated for luciferase expression in a Bruker in vivo Xtreme imaging system (Brueker, Billerica, MA, USA). Tumor growth was visualized with D-luciferin (150 mg/kg). All animals were weighed daily. Animals that lost greater than 20% of their total starting body weight were euthanized. Animal experiments were carried out under the protocol and ethics code approved by the Stony Brook University IACUC committee (protocol number: 764761-1).

## 5. Conclusions

In conclusion, we have discovered a novel mechanism by which miR-489 inhibits the growth of TNBC cells and tumors. We also laid a foundation for the development of CMM489 as a potential new agent for treatment of TNBCs based on our proof of concept in vitro and in vivo models. In future studies, we will focus on combination treatment options involving CMM489 and other therapeutic agents currently being used for breast cancer treatments. Successful completion of our studies will characterize CMM489 as a novel therapeutic option for TNBCs that currently has no effective targeted therapies.

## Supplementary Materials

The following supporting information can be downloaded at: https://www.mdpi.com/2072-6694/12/8/2209/s1, Figure S1: miR-489 mediates anti-proliferative role in TNBC cells; Figure S2: The levels of miR-489 in transfected cells; Figure S3: Full pictures of the Western blots.
