# Correction: Soung et al. Therapeutic Potential of Chemically Modified MiR-489 in Triple-Negative Breast Cancers. *Cancers* 2020, *12*, 2209

**DOI:** 10.3390/cancers16051010

**Published:** 2024-02-29

**Authors:** Young Hwa Soung, Heesung Chung, Cecilia Yan, Andrew Fesler, Hyungjin Kim, Eok-Soo Oh, Jingfang Ju, Jun Chung

**Affiliations:** 1 Department of Pathology, Renaissance School of Medicine, Stony Brook University, Stony Brook, NY 11794, USA; younghwa.song@stonybrookmedicine.edu (Y.H.S.); heesung.chung@stonybrook.edu (H.C.); cecilia.yan@stonybrook.edu (C.Y.); andrew.fesler@stonybrook.edu (A.F.); jingfang.ju@stonybrookmedicine.edu (J.J.); 2 Department of Life Sciences, Ewha Womans University, Seoul 03760, Republic of Korea; ohes@ewha.ac.kr; 3 Department of Pharmacological Sciences, Renaissance School of Medicine, Stony Brook University, Stony Brook, NY 11794, USA; hyungjin.kim@stonybrook.edu

In the original publication [1], there was a mistake in Figure 4C and the Supplementary Materials as published. GAPDH blot in Figure 3B was used in GAPDH blot in Figure 4C by mistake. We realized that the GAPDH band for Figure 4C was incorrectly labeled as β-actin in Figure S3. We herein replace the erroneous figure with the correct one. For the Supplementary Materials, we add the full pictures of the Western blots in Figure S3. The corrected GAPDH of Figure 4C and the description of the Supplementary Materials appear below. The authors apologize for any inconvenience caused and state that the scientific conclusions remain unaffected. This correction was approved by the Academic Editor. The original publication has also been updated.

**Figure 4:**
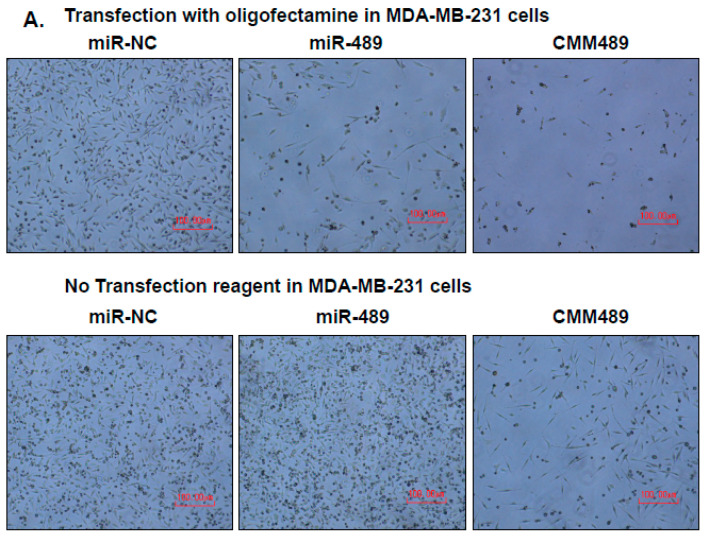

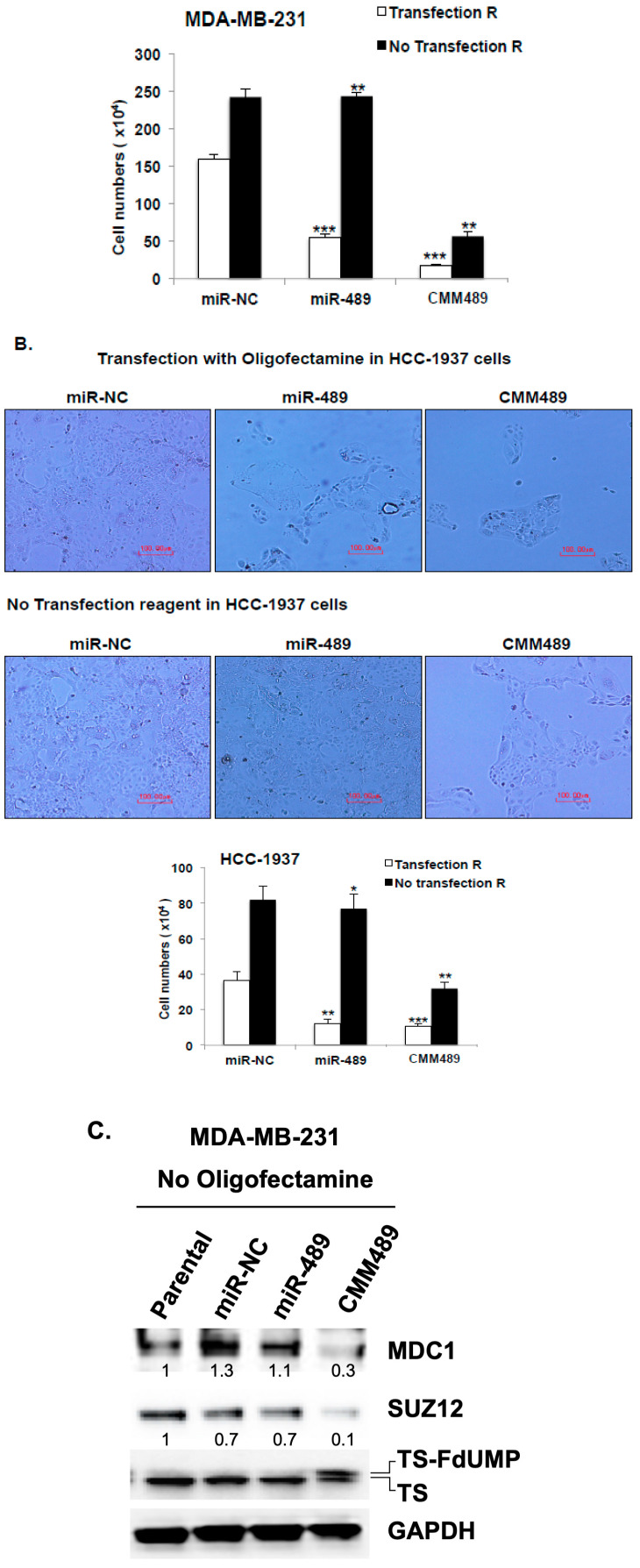
Delivery of 5-FU-miR-489 mimic (CMM489) does not require a transfection reagent. miR-negative control (NC), miR-489 and CMM489 were transfected into MDA-MB-231 cells (**A**) or HCC-1937 cells (**B**) with or without oligofectamine reagent. The density of cells was imaged at 6 days by phase contrast microscopy. Representative images were selected from three independent experiments. Scale bar: 100 μm. The cell numbers were counted using Trypan blue staining and a hematocytometer. Column, mean from three independent experiments; bars, SD. * *p* < 0.05, ** *p* < 0.01, *** *p* < 0.001. (**C**) Whole cell lysate from MDA-MB-231 cells transfected with the indicated miRNAs without oligofectamine reagent were analyzed by Western blot assay with antibodies against MDC1, SUZ12, TS-FdUMP and β-actin. Densitometric analysis was performed using Image J. Representative images were carried out at least 3 times.

**Supplementary Materials:** The following supporting information can be downloaded at: https://www.mdpi.com/2072-6694/12/8/2209/s1, Figure S1: miR-489 mediates anti-proliferative role in TNBC cells; Figure S2: The levels of miR-489 in transfected cells; Figure S3: Full pictures of the Western blots.
