# Supplementary material for: Therapeutic Potential of Chemically Modified miR-489 in Triple-Negative Breast Cancers
Source: Cancers (Basel). 2020 Aug 7;12(8):2209. doi: 10.3390/cancers12082209 (PMC7463492; doi:10.3390/cancers12082209)

Supplementary Materials

Therapeutic potential of chemically modified miR-489 in Triple Negative Breast Cancers

Young Hwa Soung[1†], Heesung Chung[1,3†], Cecilia Yan[1], Andrew Fesler[1], Hyungjin Kim[2], Eok-Soo Oh[3], Jingfang Ju[1] and Jun Chung[1*]

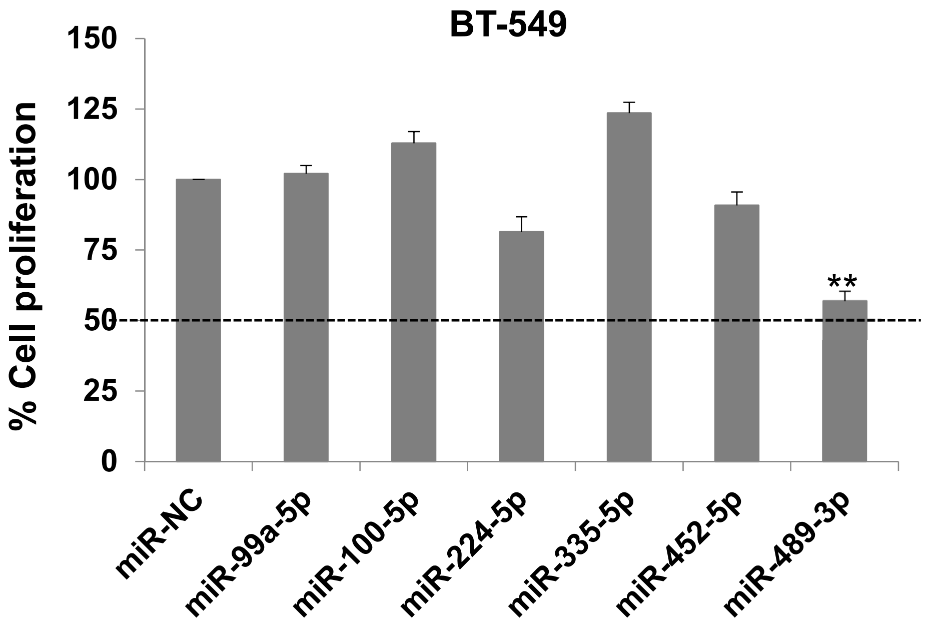

Figure S1. BT-549 cells transfected with indicated miRNA mimics were incubated for 96 hrs. Proliferation of these cells was measured by MTT assay. Data are represented as mean ± SD (n = 3). ***P < 0.01* compared with the control

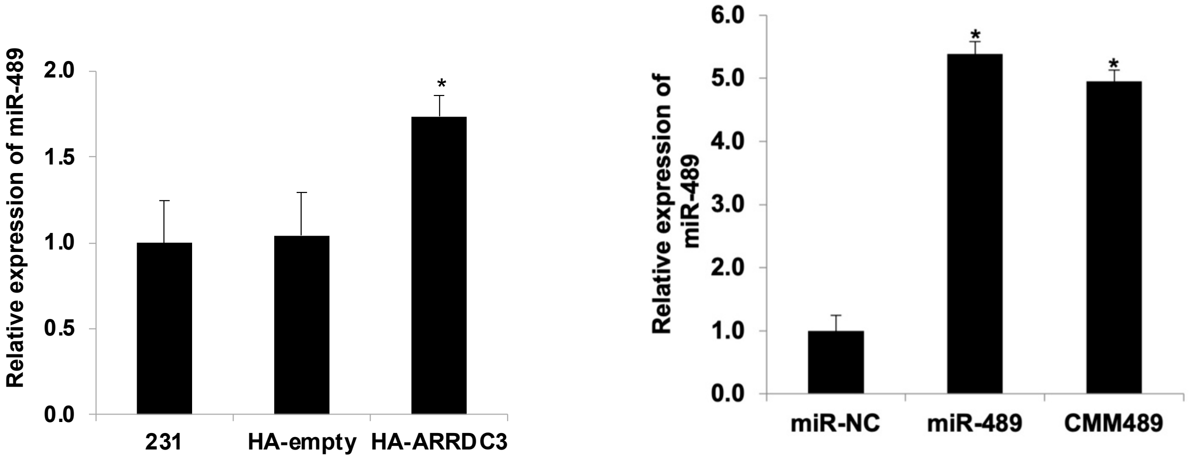

Figure S2. miR-489 expression level quantitated by qRT-PCR in MDA-MB-231 cells. RNAs were isolated from cells transfected with indicated (A) vector or (B) miRNAs. qRT-PCR was performed as described in the Material and Method section. Column, mean from at least three independent experiments; bars, SD. The statistical analysis was done using Student's *t* test. *, *P<0.05.*

Figure S3. Full pictures of the western blots. Images of band were acquired using ChemiDoc Imaging system with ImageLab software 6. 1. (Bio-Rad).

The Western blot original images for Figure 1C

MDA-MB-231

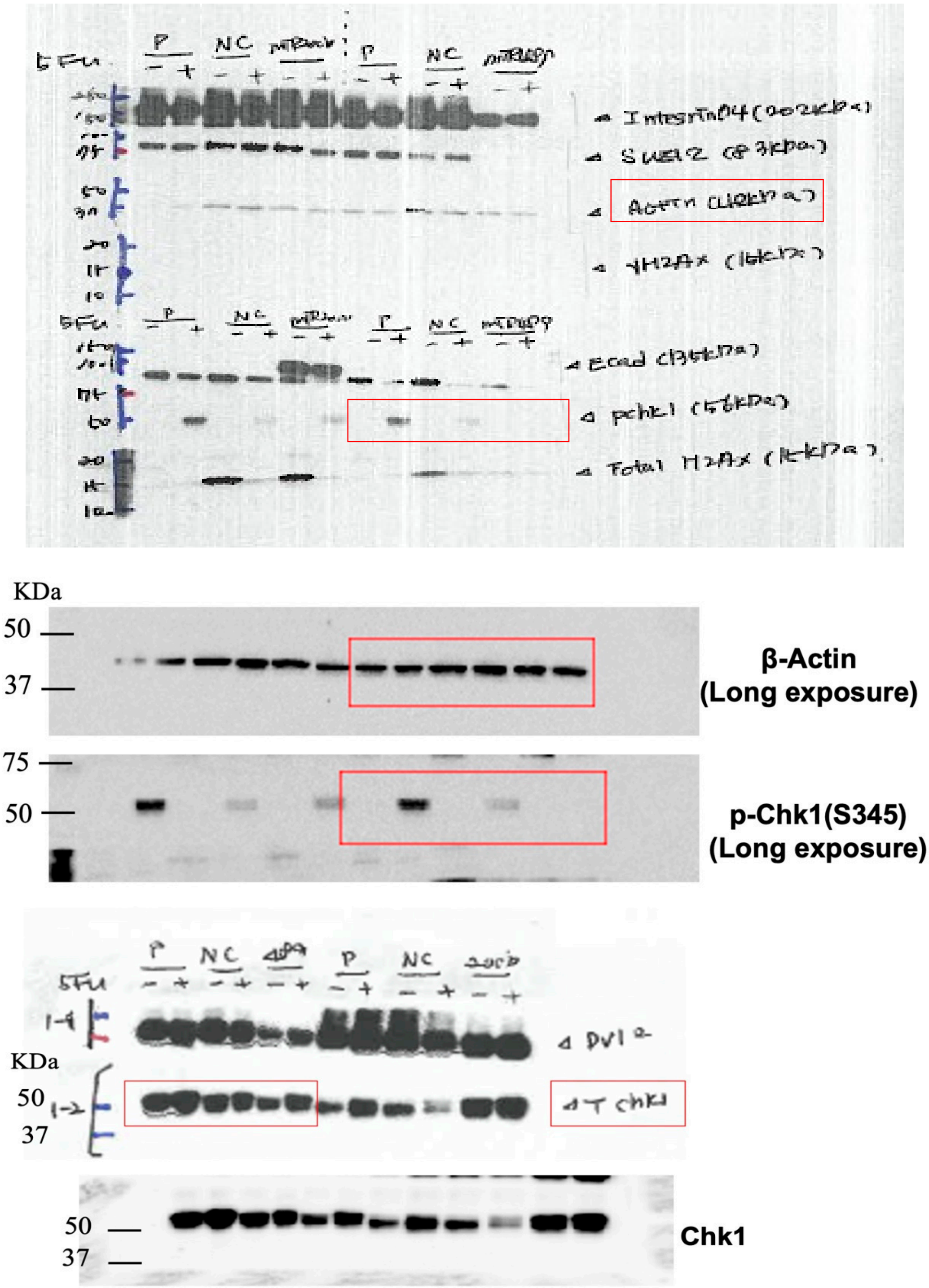

The Western blot original images for Figure 1C

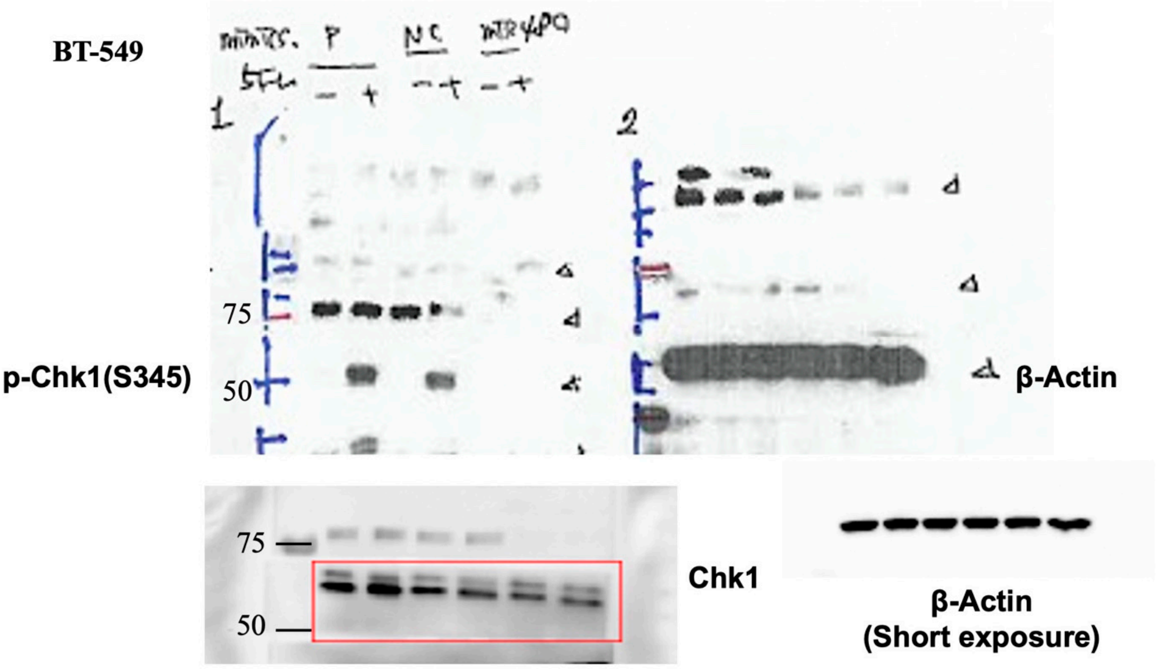

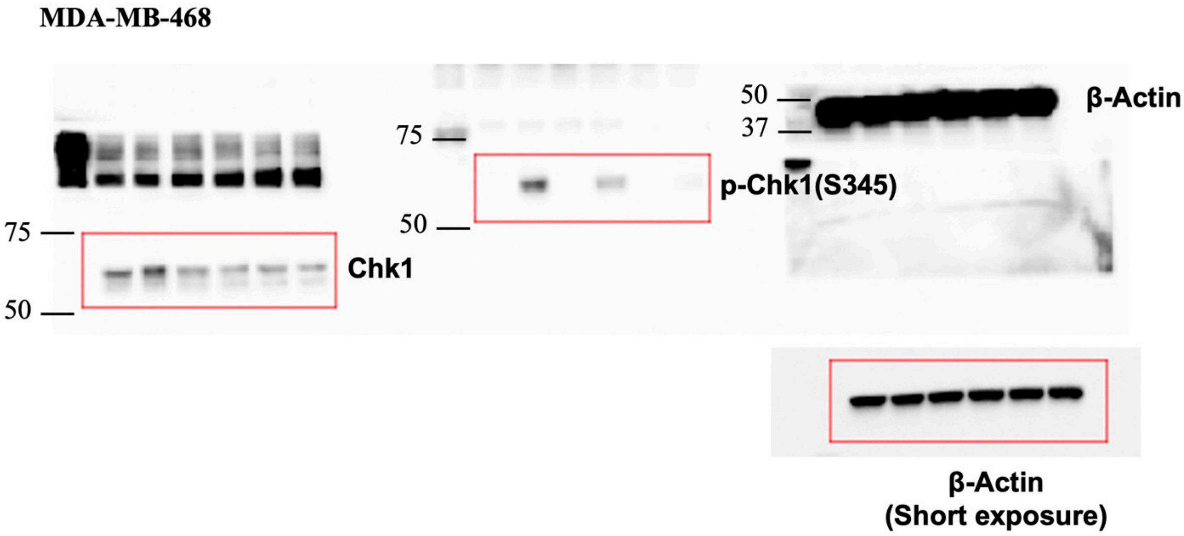

The Western blot original images for Figure 2B

MDA-MB-231

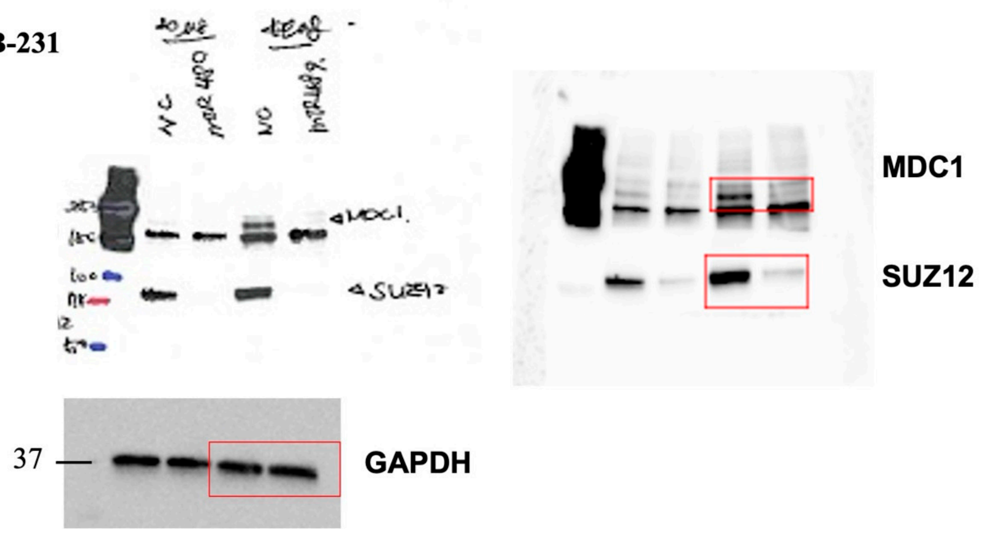

HCC-1937

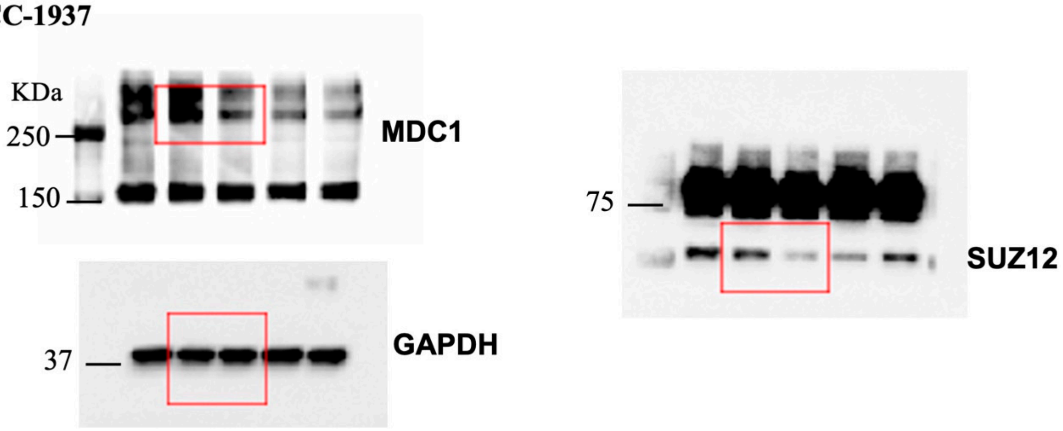

BT-549

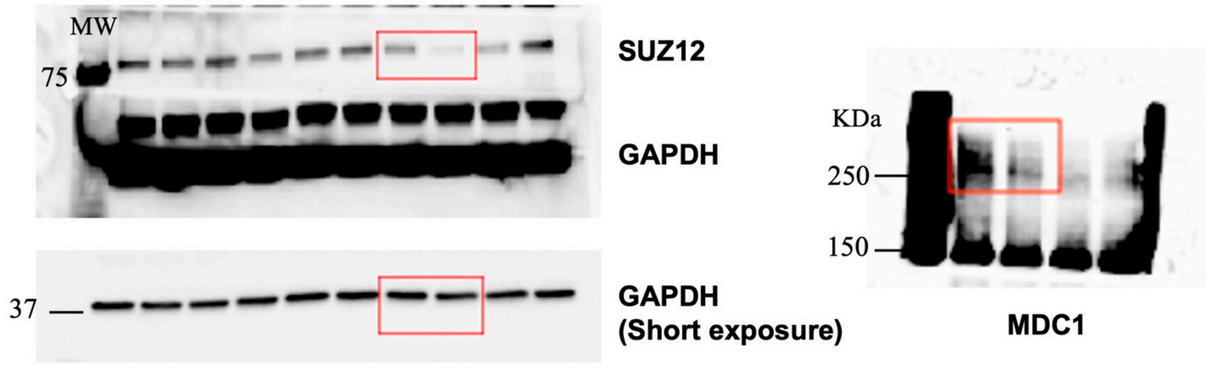

The Western blot original images for Figure 2C

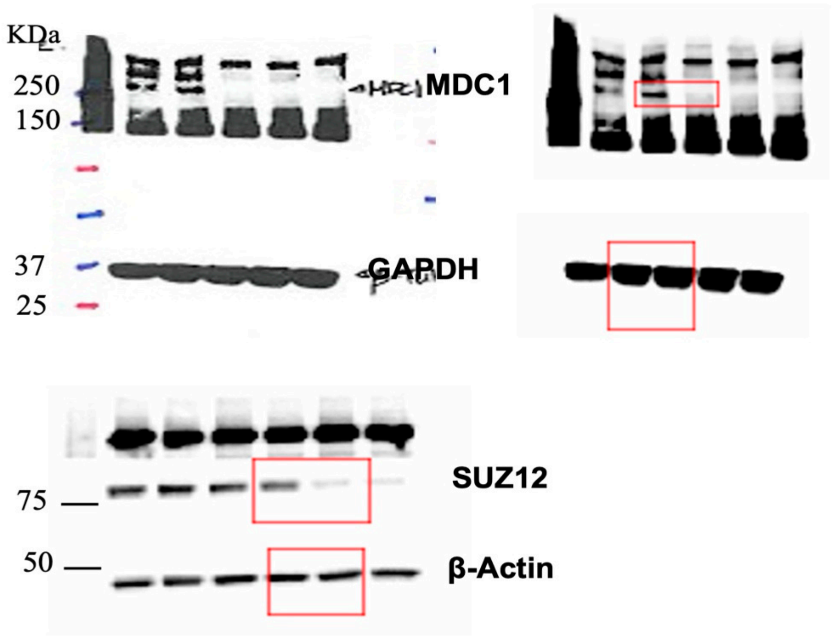

The Western blot original images for Figure 3B

MDA-MB-231

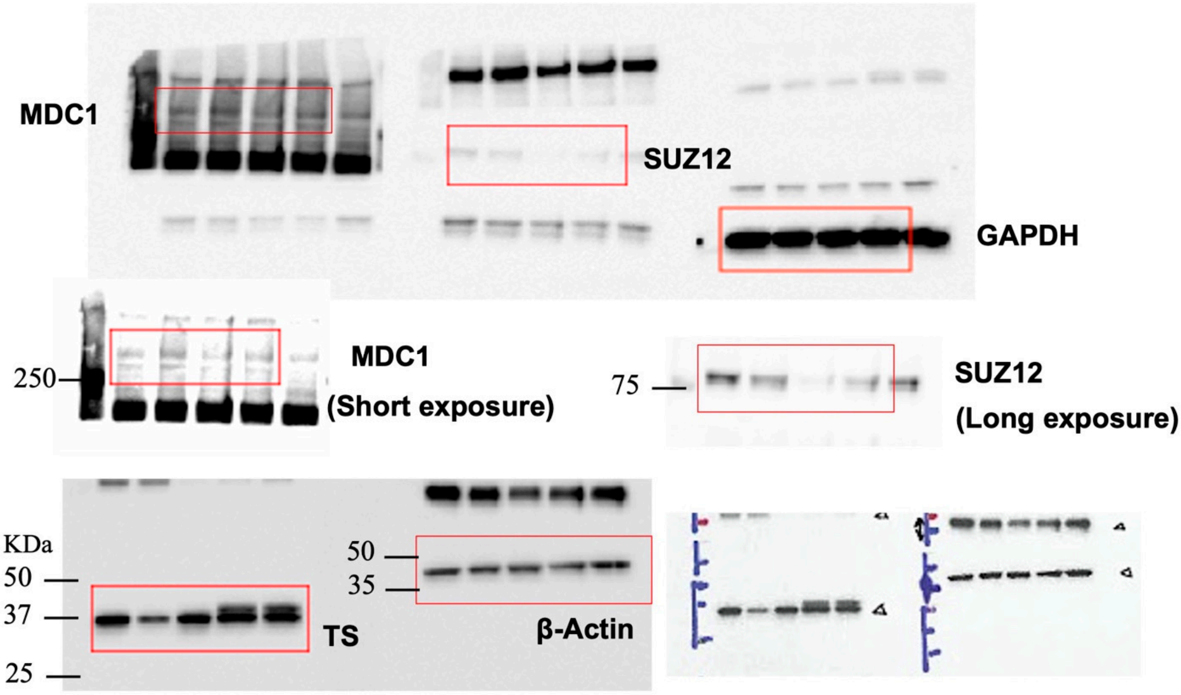

The Western blot original images for Figure 3B

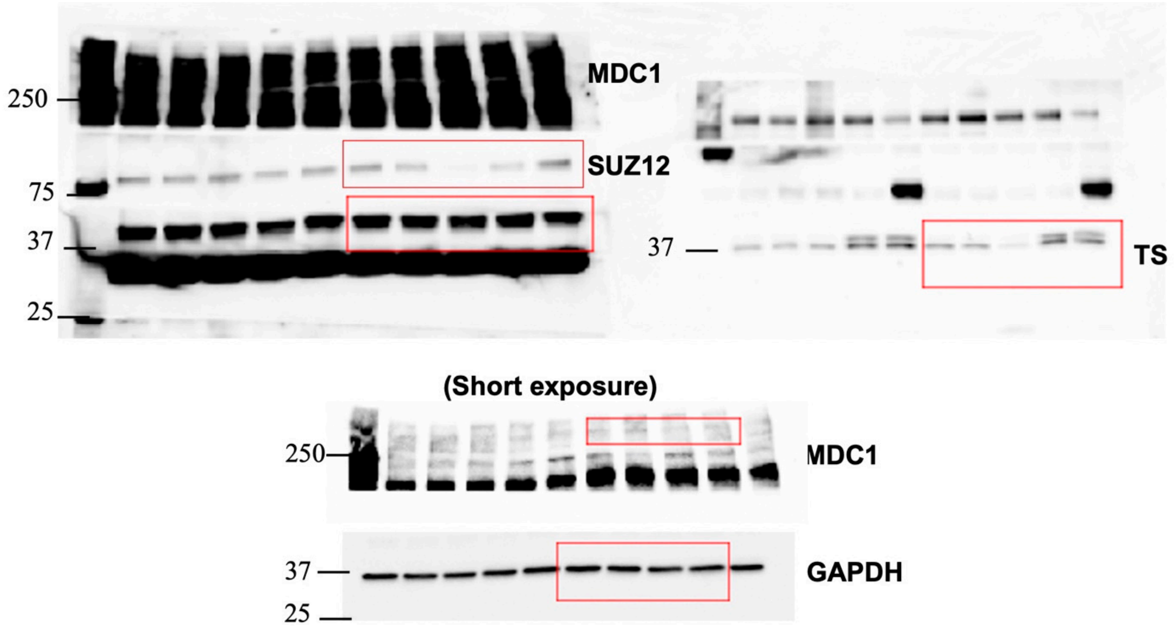

The Western blot original images for Figure 4C

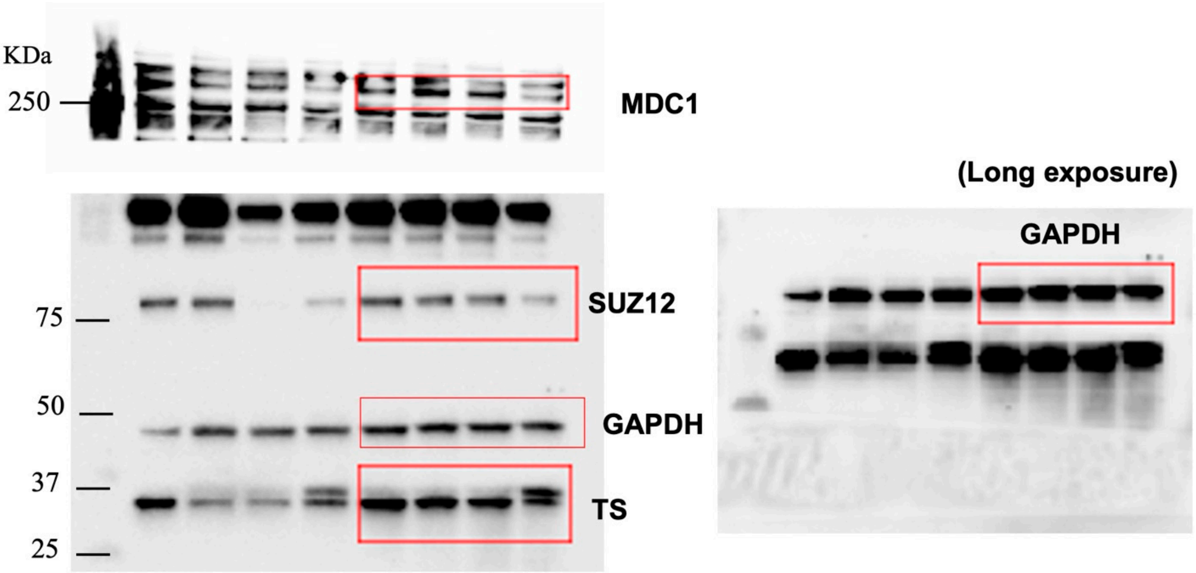

Supplement: Supplementary file 1 [file cancers-12-02209-s001.pdf]
